# On the Utility of Alkalies in the Treatment of Rheumatism

**Published:** 1849-03

**Authors:** J. I. Furnivall

**Affiliations:** Holloway


*On the utility of Alkalies in the Treatment of Rheumatism.* By. J. I. Furnivall, M.D., Holloway. — Some remarks on the treatment of rheumatism by alkalies are published in the Lancet of 25th November last. I have now, for nearly twenty years, been in the habit of treating rheumatism by means of alkalies, (the liquor potassæ, the carbonate, bicarbonate of potass or soda); and as cases have multiplied in my practice during that long period, I have become more and more satisfied of their efficacy in preventing the supervention of heart disease; while as to their value in curing rheumatism, I beg to refer to reports published about a year ago, by Dr. Wright, of Birmingham.

I have seldom used them alone in severe and threatening cases, though Dr. Wright has done so with great success; but considering that the inflammation and pyrexia were the effects or concomitants of the peculiar state of the blood in rheumatic fever, to remove which state alkalies are recommended, I have combined with the alkalies various other remedies—colchicum, to remove pain and lower excitement, mercury sometimes, etc., etc.

The results of my clinical observations have been these—

1st. That no case of supervening heart disease has ever occurred in my practice since I have administered alkalies in rheumatic cases; nor will they, in my opinion, if the concomitant inflammation and fever have at the same time been properly attended to.

2dly. That many cases of rheumatic fever are on record which have been energetically treated by medical men of eminence, but without the use of alkalies, in which heart disease has ensued, and proved fatal.

3dly. That mercury and colchicum, separate or combined, and either or both pushed to their utmost extent, will not secure the patient from heart disease, without the addition of alkalies.—*London Lancet.*

